# *catena*-Poly[2,2′,2′′-nitrilo­tris­(ethan­aminium) [tri-μ-oxido-tris­[dioxido­vanadate(V)]] monohydrate]

**DOI:** 10.1107/S1600536813026056

**Published:** 2013-10-02

**Authors:** Kelvin B. Chang, Matthew D. Smith, Matthias Zeller, Alexander J. Norquist

**Affiliations:** a Department of Chemistry, Haverford College, 370 Lancaster Avenue, Haverford, PA 19041, USA; b Department of Chemistry, Youngstown State University, 1 University Plaza, Youngstown, OH 44555, USA

## Abstract

The title compound, {(C_6_H_21_N_4_)[V_3_O_9_]·H_2_O}_*n*_, crystallizes as a salt with [trenH_3_]^3+^ cations [tren is tris­(2-amino­eth­yl)amine], and one-dimensional anionic {[V^V^O_3_]^−^}_*n*_ (metavanadate) chains along the *c*-axis direction. Three crystallographically distinct V^V^ sites and one occluded water mol­ecule are present for every [trenH_3_]^3+^ cation in the unit cell. The {[V^V^O_3_]^−^}_*n*_ chains are composed of vertex-sharing [VO_4_] tetra­hedra and have a repeat unit of six tetra­hedra. Each tetra­hedron in the chain contains two terminal and two μ^2^-bridging oxide ligands. The [trenH_3_]^3+^ cations, {[V^V^O_3_]^−^}_*n*_ anions and occluded water mol­ecules participate in an extensive three-dimensonal hydrogen-bonding network. The three terminal ammonium sites of the [trenH_3_]^3+^ cations each form strong N—H⋯O hydrogen bonds to terminal oxide ligands on the {[V^V^O_3_]^−^}_*n*_ chain. Each occluded water mol­ecule also donates two O—H⋯O hydrogen bonds to the terminal oxide ligands.

## Related literature

For properties of organically templated metal oxides, see: Cheetham *et al.* (1999 ▶). A host of amine-templated metavanadate chains with connectivities identical to the title compound have been reported previously, for examples, see: Riou & Ferey (1996 ▶); Roman *et al.* (1991 ▶); Smith *et al.* (2012 ▶). Metavanadate chains with repeat units of six tetra­hedra are known to exist, see: Lin *et al.* (2003 ▶); Tyrselová *et al.* (1995 ▶). For details of the H-atom treatment in the refinement, see: Cooper *et al.* (2010 ▶). 
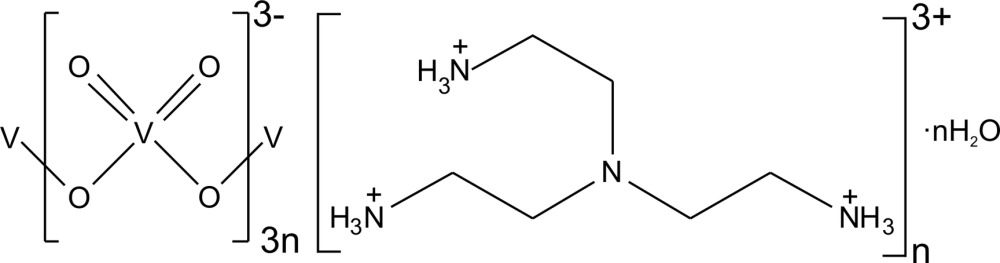


## Experimental

### 

#### Crystal data

(C_6_H_21_N_4_)[V_3_O_9_]·H_2_O
*M* *_r_* = 464.09
Monoclinic, 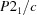

*a* = 9.6624 (14) Å
*b* = 10.9179 (15) Å
*c* = 15.768 (2) Å
β = 100.565 (2)°
*V* = 1635.2 (4) Å^3^
*Z* = 4
Mo *K*α radiation
μ = 1.73 mm^−1^
*T* = 100 K
0.26 × 0.20 × 0.16 mm

#### Data collection

Bruker SMART APEX CCD area-detector diffractometer
Absorption correction: multi-scan (*SADABS*; Bruker, 2008 ▶) *T*_min_ = 0.623, *T*_max_ = 0.746
14279 measured reflections
4719 independent reflections
3195 reflections with *I* > 2.0σ(*I*)
*R*_int_ = 0.061

#### Refinement

*R*[*F* ^2^ > 2σ(*F* ^2^)] = 0.052
*wR*(*F* ^2^) = 0.094
*S* = 1.00
2860 reflections
208 parameters
H-atom parameters not refined
Δρ_max_ = 1.44 e Å^−3^
Δρ_min_ = −0.68 e Å^−3^

### 

Data collection: *APEX2* (Bruker, 2009 ▶); cell refinement: *SAINT* (Bruker, 2009 ▶); data reduction: *SAINT*; program(s) used to solve structure: *SHELXS86* (Sheldrick, 2008 ▶); program(s) used to refine structure: *CRYSTALS* (Betteridge *et al.*, 2003 ▶); molecular graphics: *CAMERON* (Watkin *et al.*, 1996 ▶); software used to prepare material for publication: *CRYSTALS*.

## Supplementary Material

Crystal structure: contains datablock(s) global, I. DOI: 10.1107/S1600536813026056/hp2060sup1.cif

Structure factors: contains datablock(s) I. DOI: 10.1107/S1600536813026056/hp2060Isup2.hkl

Additional supplementary materials:  crystallographic information; 3D view; checkCIF report

## Figures and Tables

**Table 1:** Selected bond lengths (Å)

V1—O1	1.613 (3)
V1—O2	1.651 (3)
V1—O3	1.773 (4)
V1—O4	1.793 (3)
V2—V3	3.2585 (11)
V2—O4	1.793 (3)
V2—O5	1.654 (3)
V2—O6	1.623 (4)
V2—O7	1.778 (3)
V3—O3^i^	1.769 (4)
V3—O7	1.793 (3)
V3—O8	1.651 (4)
V3—O9	1.632 (3)

Symmetry code: (i) 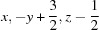.

**Table 2:** Hydrogen-bond geometry (Å, °)

*D*—H⋯*A*	*D*—H	H⋯*A*	*D*⋯*A*	*D*—H⋯*A*
O10—H1⋯O1^i^	0.95	2.06	2.997 (6)	170 (1)
O10—H2⋯O9^ii^	0.95	1.83	2.739 (6)	160 (1)
N2—H7⋯O8^iii^	0.95	1.97	2.864 (6)	155 (1)
N2—H8⋯O1^iii^	0.95	2.18	2.951 (6)	138 (1)
N2—H9⋯O5^iv^	0.95	1.98	2.850 (6)	152 (1)
C4—H13⋯O9^iii^	0.97	2.50	3.447 (6)	167 (1)
N3—H14⋯O5^iv^	0.95	2.00	2.912 (6)	162 (1)
N3—H15⋯O2^iv^	0.95	1.87	2.819 (6)	176 (1)
N3—H16⋯O6^v^	0.95	2.03	2.853 (6)	144 (1)
N3—H16⋯O10^v^	0.95	2.23	2.925 (6)	129 (1)
C6—H19⋯O6^v^	0.97	2.54	3.222 (6)	127 (1)
N4—H21⋯O4^v^	0.95	2.14	3.013 (6)	152 (1)
N4—H22⋯O2^vi^	0.95	1.94	2.796 (6)	150 (1)
N4—H23⋯O5^iv^	0.95	1.90	2.803 (6)	157 (1)

Symmetry codes: (i) 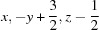; (ii) 
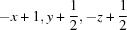
; (iii) 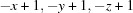; (iv) 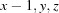; (v) 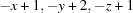; (vi) 
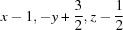
.

**Fig. 1.**
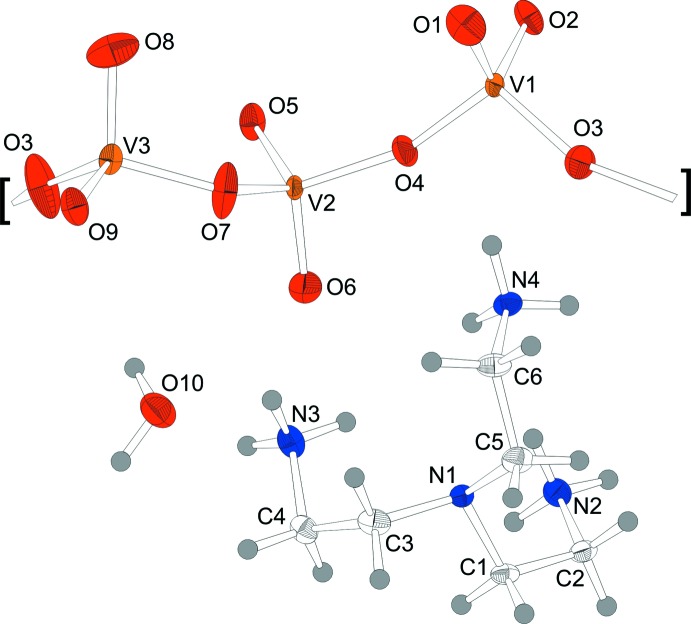
The title compound with displacement ellipsoids drawn at the 50% probability level. H atoms are shown as spheres of arbitary radius.

**Fig. 2.**
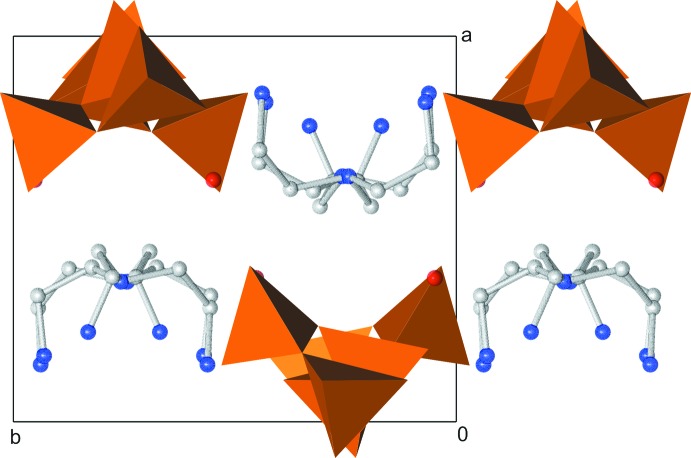
A packing diagram of the title compound, viewed down the c axis. Orange tetrahedra represent [VO4], while red, blue and white spheres represent oxygen, nitrogen, carbon and hydrogen atoms, respectively.

## supplementary crystallographic information

### 1. Comment

Organically templated metal oxides have been the subject of sustained interest for many years, owing to their structural diversity and potential to exhibit technologically desirable physical properties (Cheetham *et al.*, 1999). Amine-templated metavanadate chain compounds are no exception, with a number of different topologies for chains with identical connectivity having been reported. The synthesis and crystal structure of the title compound, [trenH_3_][VO_3_]_3_·H_2_O, is described here. The three-dimensional packing of the title compound is shown in Fig. 2. The {[V^V^O_3_]^-^}*_n_* chains are composed of vertex-sharing [VO_4_] tetrahedra and have a repeat unit of six tetrahedra. Each tetrahedron in the chain contains two terminal and two µ^2^-bridging oxide ligands. The V – O_terminal_ and V – O_bridging_ bond lengths range from 1.613 (4) to 1.653 (3) Å and 1.770 (4) to 1.794 (4) Å, respectively. Several compounds containing amine templated metavanadate chains have been previously reported, see: Riou *et al.* (1996), Roman *et al.* (1991), Smith *et al.* (2012), Lin *et al.* (2003) and Tyrselová *et al.* (1995). However, the chain topology of the inorganic anion in the title compound is novel. The [trenH_3_]^3+^ cations, {[V^V^O_3_]^-^}*_n_* anions and occluded water molecules participate in an extensive three-dimensonal hydrogen-bonding network. The three terminal ammonium sites of the [trenH_3_]^3+^ cations each form strong N–H···O hydrogen bonds to terminal oxide atoms on the {[V^V^O_3_]^-^}*_n_* chain, with N···O distances ranging from 2.796 (5) to 3.013 (5) Å. Each occluded water molecule also donates two O–H···O hydrogen bonds to the terminal oxide atoms, with O···O distances ranging from 2.738 (4) to 2.996 (5) Å.

### 2. Experimental

All chemicals (reagent grade) were commercially available and were used as received. Deionized water was used in this synthesis. The title compound was prepared under mild hydrothermal conditions in a 23 mL Teflon-lined stainless-steel autoclave. V_2_O_5_ (0.1449 g, 0.7967 mmol), Na_2_TeO_3_ (0.0889 g, 0.4012 mmol), tren (0.0620 g, 0.4240 mmol), ethanol (4 ml, 0.069 mol), and H_2_O (4 ml, 0.22 mol) were mixed to give a reaction mixture with a molar composition ratio of 2:1:1:170:550. The role of Na_2_TeO_3_ in this reaction is not understood. Reactions were placed in a 110 °C oven for 5 d. The reactions were then cooled to room temperature at a rate of 6 °C h^-1^ to promote the growth of large single crystals. Reaction vessels were opened in air, and the title compound was recovered as clear colorless needles in the presence of an unidentified tan powder *via* vacuum filtration.

### 3. Refinement

While the H atoms were all located in a difference map, they were repositioned geometrically. The H atoms were initially refined with soft restraints on the bond lengths and angles to regularize their geometry (C—H in the range 0.93–0.98, N—H to 0.95, and O—H = 0.95 Å) and *U*_iso_(H) (in the range 1.2–1.5 times *U*_eq_ of the parent atom), after which the positions were refined with riding constraints (Cooper *et al.*, 2010).

### Crystal data

**Table:**

(C_6_H_21_N_4_)[V_3_O_9_]·H_2_O	*F*(000) = 944
*M**_r_* = 464.09	*D*_x_ = 1.885 Mg m^−^^3^
Monoclinic, *P*2_1_/*c*	Mo *K*α radiation, λ = 0.71073 Å
Hall symbol: -P 2ybc	Cell parameters from 2067 reflections
*a* = 9.6624 (14) Å	θ = 2.6–29.5°
*b* = 10.9179 (15) Å	µ = 1.73 mm^−^^1^
*c* = 15.768 (2) Å	*T* = 100 K
β = 100.565 (2)°	Block, colorless
*V* = 1635.2 (4) Å^3^	0.26 × 0.20 × 0.16 mm
*Z* = 4	

### Data collection

**Table:**

Bruker SMART APEX CCD area-detector diffractometer	4719 independent reflections
Radiation source: fine focus sealed tube	3195 reflections with *I* > 2.0σ(*I*)
Graphite monochromator	*R*_int_ = 0.061
ω scans	θ_max_ = 30.1°, θ_min_ = 2.1°
Absorption correction: multi-scan (*SADABS*; Bruker, 2008)	*h* = −13→13
*T*_min_ = 0.623, *T*_max_ = 0.746	*k* = −15→15
14279 measured reflections	*l* = −20→22

### Refinement

**Table:**

Refinement on *F*^2^	Primary atom site location: structure-invariant direct methods
Least-squares matrix: full	Hydrogen site location: inferred from neighbouring sites
*R*[*F*^2^ > 2σ(*F*^2^)] = 0.052	H-atom parameters not refined
*wR*(*F*^2^) = 0.094	Method = Modified Sheldrick *w* = 1/[σ^2^(*F*^2^) + (0.03*P*)^2^ + 7.47*P*] , where *P* = (max(*F*_o_^2^,0) + 2*F*_c_^2^)/3
*S* = 1.00	(Δ/σ)_max_ = 0.0003744
2860 reflections	Δρ_max_ = 1.44 e Å^−^^3^
208 parameters	Δρ_min_ = −0.68 e Å^−^^3^
0 restraints	

### Fractional atomic coordinates and isotropic or equivalent isotropic displacement parameters (Å^2^)

**Table:**

	*x*	*y*	*z*	*U*_iso_*/*U*_eq_	
V1	0.92597 (7)	0.75226 (7)	0.71937 (5)	0.0135	
V2	0.87185 (7)	0.80317 (7)	0.49412 (4)	0.0120	
V3	0.75379 (8)	0.58481 (7)	0.35925 (5)	0.0152	
O1	0.8914 (4)	0.6089 (3)	0.7288 (2)	0.0320	
O2	1.0944 (3)	0.7734 (3)	0.7595 (2)	0.0217	
O3	0.8219 (5)	0.8480 (4)	0.7734 (2)	0.0498	
O4	0.8935 (3)	0.8036 (3)	0.6095 (2)	0.0244	
O5	1.0270 (3)	0.7839 (3)	0.4659 (2)	0.0220	
O6	0.8112 (4)	0.9374 (3)	0.4625 (2)	0.0379	
O7	0.7512 (4)	0.6883 (4)	0.4472 (2)	0.0354	
O8	0.8542 (4)	0.4695 (4)	0.4011 (3)	0.0429	
O9	0.5943 (3)	0.5335 (3)	0.3264 (2)	0.0233	
O10	0.6272 (4)	0.9517 (3)	0.2942 (2)	0.0326	
N1	0.3633 (3)	0.7571 (3)	0.4750 (2)	0.0124	
N2	0.1483 (4)	0.5617 (3)	0.4189 (2)	0.0186	
N3	0.2327 (4)	0.8310 (4)	0.6220 (2)	0.0199	
N4	0.1714 (4)	0.9305 (3)	0.3650 (2)	0.0173	
C1	0.3965 (4)	0.6257 (4)	0.4704 (3)	0.0144	
C2	0.2953 (4)	0.5559 (4)	0.4031 (3)	0.0161	
C3	0.4414 (4)	0.8077 (4)	0.5560 (3)	0.0180	
C4	0.3751 (4)	0.7766 (4)	0.6334 (3)	0.0178	
C5	0.4008 (5)	0.8229 (4)	0.4007 (3)	0.0195	
C6	0.3272 (4)	0.9449 (4)	0.3853 (3)	0.0186	
H1	0.7169	0.9334	0.2807	0.0389*	
H2	0.5660	0.9794	0.2437	0.0389*	
H3	0.4912	0.6178	0.4588	0.0191*	
H4	0.3936	0.5894	0.5259	0.0187*	
H5	0.2977	0.5904	0.3470	0.0191*	
H6	0.3247	0.4713	0.4050	0.0189*	
H7	0.1445	0.5268	0.4736	0.0240*	
H8	0.0880	0.5172	0.3752	0.0240*	
H9	0.1185	0.6447	0.4180	0.0240*	
H10	0.5379	0.7765	0.5656	0.0209*	
H11	0.4435	0.8963	0.5511	0.0211*	
H12	0.4324	0.8092	0.6858	0.0219*	
H13	0.3682	0.6886	0.6390	0.0222*	
H14	0.1764	0.7994	0.5709	0.0245*	
H15	0.1904	0.8116	0.6701	0.0245*	
H16	0.2399	0.9174	0.6173	0.0245*	
H17	0.3702	0.7723	0.3497	0.0240*	
H18	0.5027	0.8342	0.4091	0.0241*	
H19	0.3508	0.9958	0.4366	0.0226*	
H20	0.3573	0.9845	0.3373	0.0229*	
H21	0.1284	1.0088	0.3561	0.0223*	
H22	0.1465	0.8828	0.3140	0.0223*	
H23	0.1400	0.8905	0.4115	0.0223*	

### Atomic displacement parameters (Å^2^)

**Table:**

	*U*^11^	*U*^22^	*U*^33^	*U*^12^	*U*^13^	*U*^23^
V1	0.0147 (3)	0.0181 (4)	0.0087 (3)	0.0034 (3)	0.0046 (3)	−0.0010 (3)
V2	0.0124 (3)	0.0158 (4)	0.0081 (3)	0.0005 (3)	0.0028 (3)	−0.0025 (3)
V3	0.0125 (3)	0.0188 (4)	0.0157 (4)	−0.0053 (3)	0.0062 (3)	−0.0037 (3)
O1	0.032 (2)	0.025 (2)	0.036 (2)	−0.0146 (15)	−0.0010 (17)	0.0016 (16)
O2	0.0239 (17)	0.0250 (18)	0.0149 (16)	−0.0088 (13)	0.0000 (13)	0.0037 (13)
O3	0.058 (3)	0.074 (3)	0.019 (2)	0.042 (2)	0.0113 (19)	−0.001 (2)
O4	0.0225 (16)	0.039 (2)	0.0122 (15)	0.0070 (15)	0.0038 (13)	0.0007 (15)
O5	0.0161 (15)	0.034 (2)	0.0177 (16)	−0.0063 (13)	0.0081 (13)	−0.0052 (14)
O6	0.056 (3)	0.031 (2)	0.025 (2)	0.0182 (19)	0.0041 (18)	0.0015 (16)
O7	0.032 (2)	0.049 (2)	0.031 (2)	−0.0281 (18)	0.0210 (17)	−0.0238 (18)
O8	0.028 (2)	0.032 (2)	0.071 (3)	0.0053 (17)	0.014 (2)	0.003 (2)
O9	0.0206 (16)	0.0303 (19)	0.0184 (17)	−0.0104 (14)	0.0023 (13)	−0.0028 (14)
O10	0.0283 (19)	0.047 (2)	0.0227 (19)	0.0063 (17)	0.0044 (15)	0.0061 (17)
N1	0.0124 (16)	0.0120 (16)	0.0136 (17)	0.0000 (14)	0.0043 (13)	0.0019 (14)
N2	0.0161 (18)	0.0198 (19)	0.0188 (19)	−0.0023 (15)	0.0007 (15)	−0.0005 (16)
N3	0.0159 (18)	0.028 (2)	0.0158 (19)	−0.0045 (16)	0.0041 (15)	0.0007 (16)
N4	0.0143 (17)	0.0140 (18)	0.022 (2)	0.0004 (14)	0.0004 (15)	−0.0020 (16)
C1	0.0109 (19)	0.015 (2)	0.018 (2)	0.0030 (16)	0.0043 (16)	0.0040 (17)
C2	0.015 (2)	0.015 (2)	0.019 (2)	0.0045 (16)	0.0043 (17)	0.0012 (17)
C3	0.0107 (19)	0.019 (2)	0.024 (2)	−0.0026 (17)	0.0016 (17)	0.0026 (19)
C4	0.016 (2)	0.017 (2)	0.018 (2)	−0.0043 (16)	−0.0014 (17)	−0.0005 (17)
C5	0.015 (2)	0.024 (2)	0.021 (2)	0.0039 (18)	0.0090 (18)	0.0054 (19)
C6	0.015 (2)	0.014 (2)	0.027 (2)	0.0009 (17)	0.0046 (18)	0.0065 (19)

### Geometric parameters (Å, º)

**Table:**

V1—O1	1.613 (3)	N3—H14	0.950
V1—O2	1.651 (3)	N3—H15	0.950
V1—O3	1.773 (4)	N3—H16	0.950
V1—O4	1.793 (3)	N4—C6	1.489 (5)
V2—V3	3.2585 (11)	N4—H21	0.950
V2—O4	1.793 (3)	N4—H22	0.950
V2—O5	1.654 (3)	N4—H23	0.950
V2—O6	1.623 (4)	C1—C2	1.510 (6)
V2—O7	1.778 (3)	C1—H3	0.969
V3—O3^i^	1.769 (4)	C1—H4	0.966
V3—O7	1.793 (3)	C2—H5	0.967
V3—O8	1.651 (4)	C2—H6	0.966
V3—O9	1.632 (3)	C3—C4	1.517 (6)
O10—H1	0.950	C3—H10	0.978
O10—H2	0.950	C3—H11	0.970
N1—C1	1.475 (5)	C4—H12	0.975
N1—C3	1.468 (5)	C4—H13	0.968
N1—C5	1.475 (5)	C5—C6	1.508 (6)
N2—C2	1.488 (5)	C5—H17	0.976
N2—H7	0.950	C5—H18	0.977
N2—H8	0.950	C6—H19	0.974
N2—H9	0.950	C6—H20	0.962
N3—C4	1.479 (6)		
			
O1—V1—O2	107.86 (17)	H15—N3—H16	109.5
O1—V1—O3	112.5 (2)	C6—N4—H21	109.5
O2—V1—O3	109.83 (19)	C6—N4—H22	109.2
O1—V1—O4	112.94 (18)	H21—N4—H22	109.5
O2—V1—O4	108.21 (15)	C6—N4—H23	109.6
O3—V1—O4	105.41 (17)	H21—N4—H23	109.5
V3—V2—O4	128.76 (12)	H22—N4—H23	109.5
V3—V2—O5	87.55 (11)	N1—C1—C2	114.0 (3)
O4—V2—O5	109.31 (15)	N1—C1—H3	108.5
V3—V2—O6	113.39 (14)	C2—C1—H3	109.6
O4—V2—O6	106.12 (17)	N1—C1—H4	108.2
O5—V2—O6	109.05 (19)	C2—C1—H4	107.9
V3—V2—O7	24.27 (11)	H3—C1—H4	108.6
O4—V2—O7	111.67 (17)	C1—C2—N2	112.1 (3)
O5—V2—O7	110.85 (16)	C1—C2—H5	109.0
O6—V2—O7	109.7 (2)	N2—C2—H5	108.7
V2—V3—O3^i^	93.50 (13)	C1—C2—H6	108.2
V2—V3—O7	24.06 (10)	N2—C2—H6	108.7
O3^i^—V3—O7	112.99 (19)	H5—C2—H6	110.2
V2—V3—O8	100.80 (15)	N1—C3—C4	112.9 (3)
O3^i^—V3—O8	110.4 (2)	N1—C3—H10	109.0
O7—V3—O8	105.2 (2)	C4—C3—H10	109.2
V2—V3—O9	130.93 (12)	N1—C3—H11	108.7
O3^i^—V3—O9	111.19 (18)	C4—C3—H11	107.8
O7—V3—O9	108.37 (16)	H10—C3—H11	109.1
O8—V3—O9	108.46 (19)	C3—C4—N3	109.4 (4)
V1—O3—V3^ii^	159.3 (2)	C3—C4—H12	110.0
V2—O4—V1	161.2 (2)	N3—C4—H12	109.1
V3—O7—V2	131.66 (19)	C3—C4—H13	110.0
H1—O10—H2	109.5	N3—C4—H13	109.4
C1—N1—C3	109.1 (3)	H12—C4—H13	108.8
C1—N1—C5	110.3 (3)	N1—C5—C6	112.4 (3)
C3—N1—C5	110.2 (3)	N1—C5—H17	107.3
C2—N2—H7	109.4	C6—C5—H17	107.7
C2—N2—H8	109.6	N1—C5—H18	110.0
H7—N2—H8	109.5	C6—C5—H18	110.1
C2—N2—H9	109.5	H17—C5—H18	109.2
H7—N2—H9	109.5	C5—C6—N4	111.7 (3)
H8—N2—H9	109.5	C5—C6—H19	109.8
C4—N3—H14	109.4	N4—C6—H19	108.2
C4—N3—H15	109.9	C5—C6—H20	108.8
H14—N3—H15	109.5	N4—C6—H20	108.5
C4—N3—H16	109.1	H19—C6—H20	109.8
H14—N3—H16	109.5		

Symmetry codes: (i) *x*, −*y*+3/2, *z*−1/2; (ii) *x*, −*y*+3/2, *z*+1/2.

### Hydrogen-bond geometry (Å, º)

**Table:**

*D*—H···*A*	*D*—H	H···*A*	*D*···*A*	*D*—H···*A*
O10—H1···O1^i^	0.95	2.06	2.997 (6)	170 (1)
O10—H2···O9^iii^	0.95	1.83	2.739 (6)	160 (1)
N2—H7···O8^iv^	0.95	1.97	2.864 (6)	155 (1)
N2—H8···O1^iv^	0.95	2.18	2.951 (6)	138 (1)
N2—H9···O5^v^	0.95	1.98	2.850 (6)	152 (1)
C4—H13···O9^iv^	0.97	2.50	3.447 (6)	167 (1)
N3—H14···O5^v^	0.95	2.00	2.912 (6)	162 (1)
N3—H15···O2^v^	0.95	1.87	2.819 (6)	176 (1)
N3—H16···O6^vi^	0.95	2.03	2.853 (6)	144 (1)
N3—H16···O10^vi^	0.95	2.23	2.925 (6)	129 (1)
C6—H19···O6^vi^	0.97	2.54	3.222 (6)	127 (1)
N4—H21···O4^vi^	0.95	2.14	3.013 (6)	152 (1)
N4—H22···O2^vii^	0.95	1.94	2.796 (6)	150 (1)
N4—H23···O5^v^	0.95	1.90	2.803 (6)	157 (1)

Symmetry codes: (i) *x*, −*y*+3/2, *z*−1/2; (iii) −*x*+1, *y*+1/2, −*z*+1/2; (iv) −*x*+1, −*y*+1, −*z*+1; (v) *x*−1, *y*, *z*; (vi) −*x*+1, −*y*+2, −*z*+1; (vii) *x*−1, −*y*+3/2, *z*−1/2.
